# Supplementary figures and images for: Pre-existing antibodies predict protection while mucosal inflammation correlates with symptomatic Bordetella pertussis infection
Source: medRxiv. 2026 Jul 9:2026.06.26.26356067. Preprint. [Version 1] doi: 10.64898/2026.06.26.26356067 (PMC13370606; doi:10.64898/2026.06.26.26356067)

Figure S1

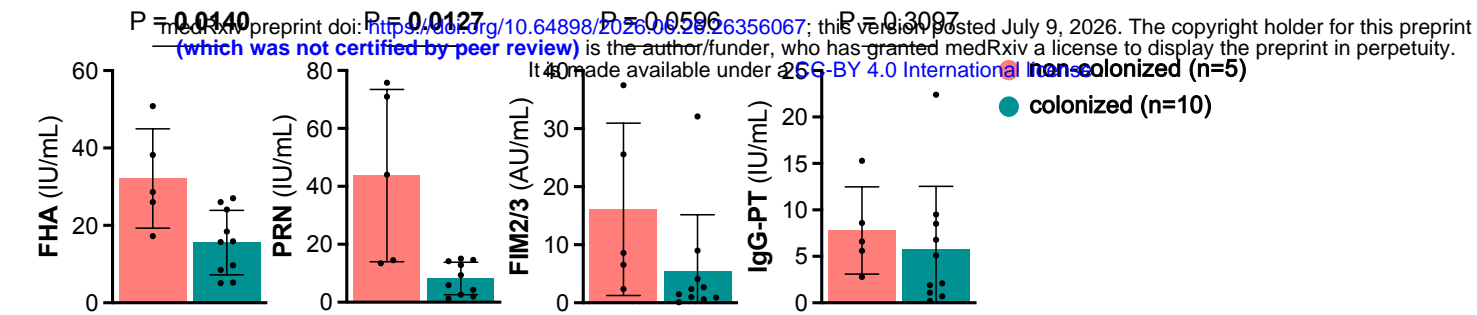

Figure S2

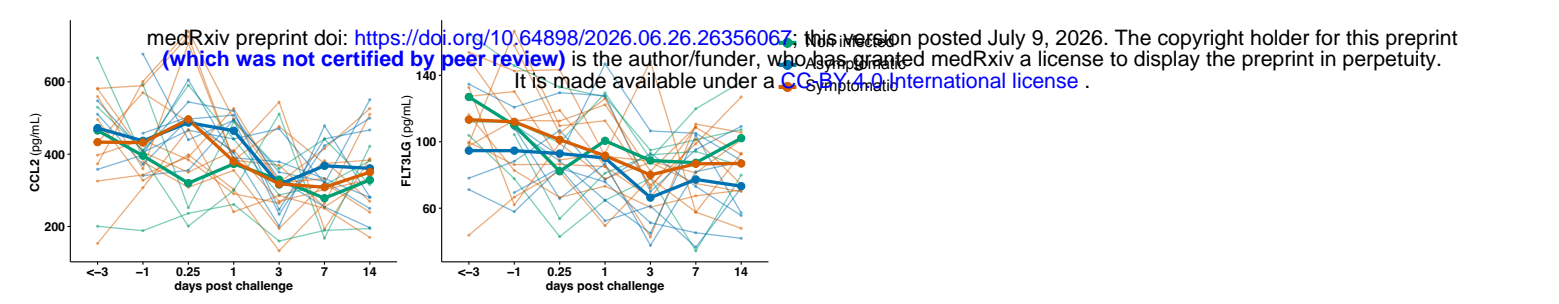



Figure S4

A

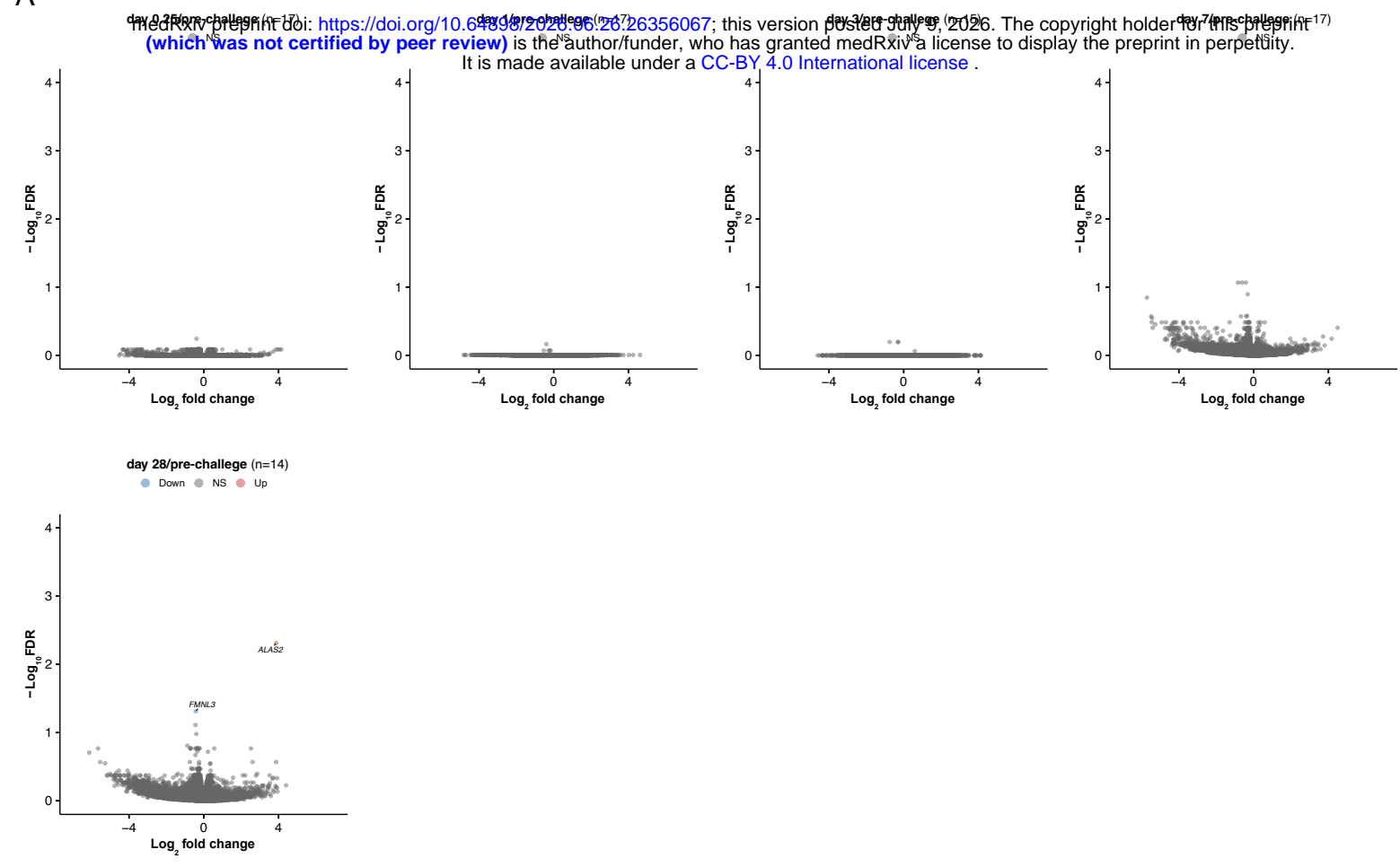

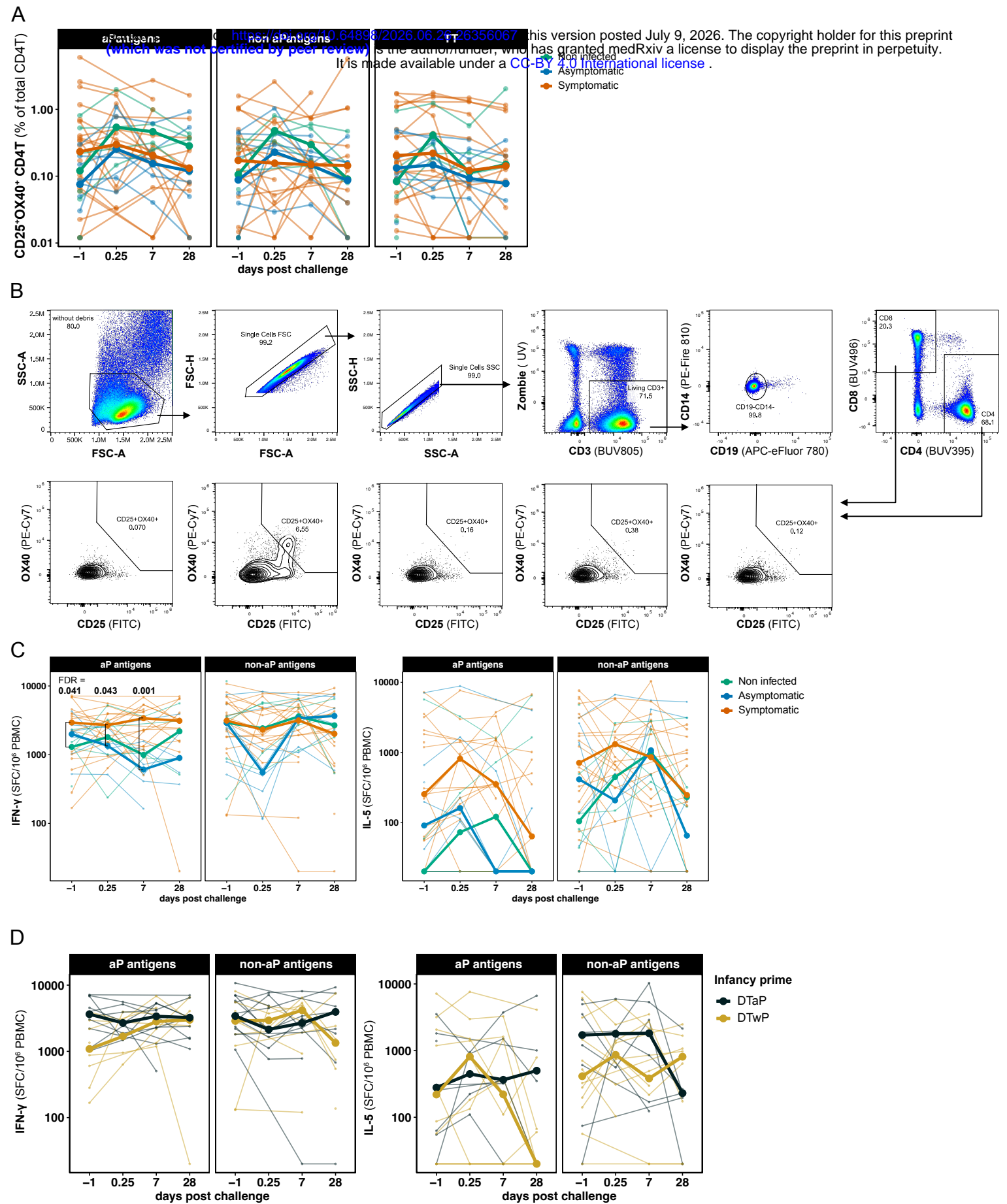

A

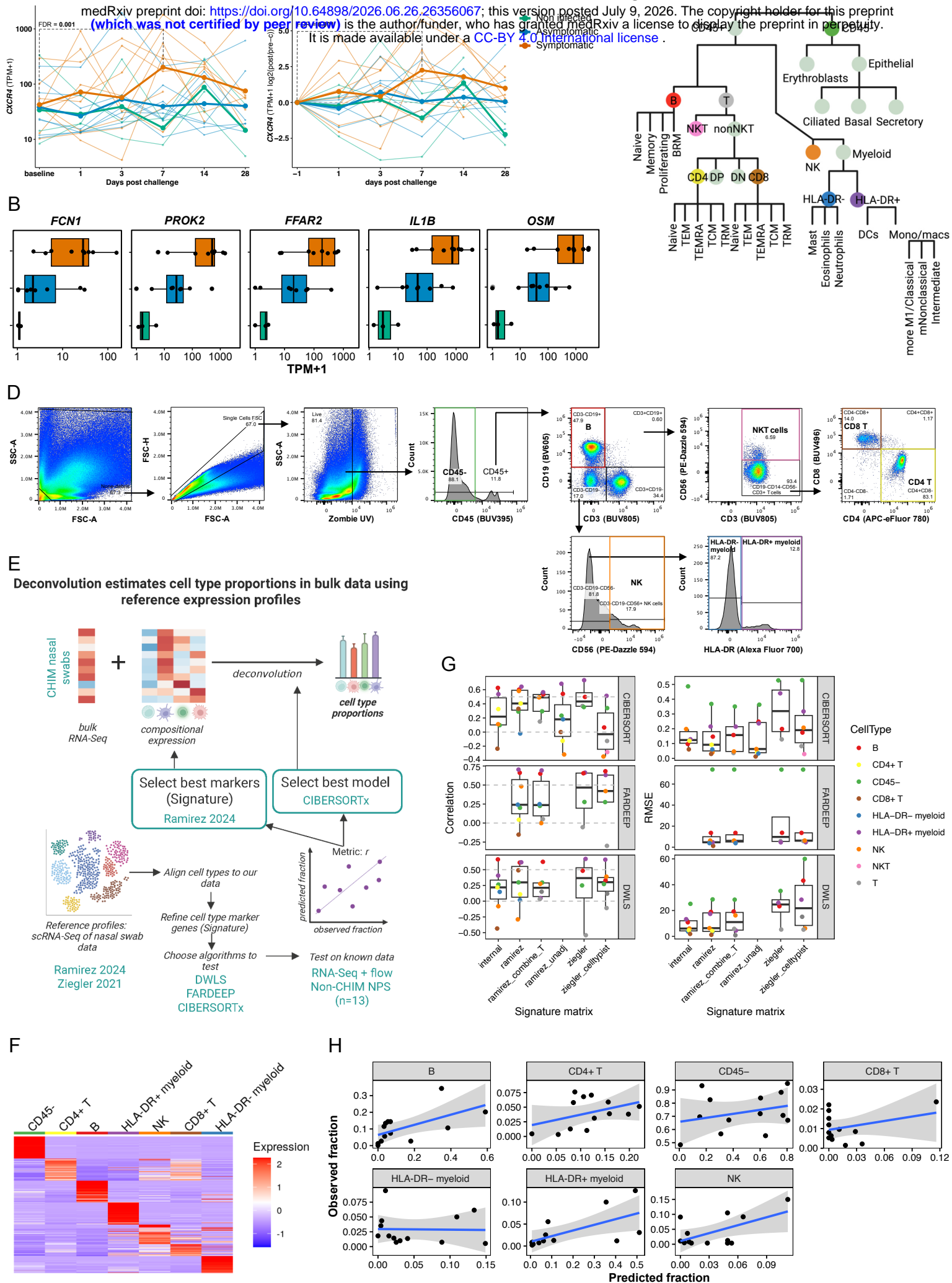

Supplement: 1 — Figure S1. Non-colonized (non-infected) participants from the PERISCOPE Bp CHIM study had higher baseline Bp-specific IgG titers. Serum IgG titers against Bp antigens (PT, PRN, FHA, FIM) before Bp inoculation per outcome definition. Mean bars with SD are depicted per outcome definition. P values were calculated by performing Mann-Whitney tests. Raw data were derived from the PERISCOPE Bp CHIM study. Figure S2. Bp challenge decreased systemic mediators independent of clinical outcome. Plasma CCL2 and FLT3LG concentrations (pg/mL) over time and coloured per outcome definition. Mean lines are shown per outcome group. Figure S3. Dynamic and clinically associated changes in blood immune cell frequencies following Bp challenge. A) PBMC subset frequencies of CD19-CD3- (left) and classical monocytes (right) over time. Mean lines are depicted per outcome definition. B) Flow cytometry gating strategy of PBMC immune cell subsets. Figure S4. Limited transcriptional changes in PBMCs following Bp challenge. Volcano plots showing the log2(fold change) and −log10(FDR) of the DEG in PBMCs of symptomatic people on day 0.25, 1, 3, 7, and 28 vs. pre-challenge with downregulated genes in blue and upregulated genes in red (FDR < 0.05). Figure S5. Bp exposure and infection did not affect antigen-specific T cell activation and polarization in the blood. A) Activation-induced marker (AIM) assays: PBMCs were stimulated with 1 μg/mL of either the pertussis (containing aP or non-aP Bp vaccine antigens) or tetanus peptide megapools for 18–24 h. CD25+OX40+ CD4 T cells were measured by flow cytometry and expressed as a percentage of total CD4 T cells, normalized to DMSO responses. Mean lines are depicted by clinical outcome; P values were calculated by multiple two-tailed Wilcoxon matched-pairs tests. B) Flow cytometry gating strategy for CD25+OX40+ CD4+ or CD8+ T cells. C-D) IFN-γ or IL-5 producing cells (spot-forming cells, SFC) were measured by Fluorospot following 14-day stimulation with aP o [file NIHPP2026.06.26.26356067V1-supplement-1.pdf]
